# Clinical relevance of single nucleotide polymorphisms within the 13 cytokine genes in North Indian trauma hemorrhagic shock patients

**DOI:** 10.1186/s13049-015-0174-3

**Published:** 2015-11-11

**Authors:** Dablu Lal Gupta, Predeep Kumar Nagar, Vineet Kumar Kamal, Sanjeev Bhoi, D. N. Rao

**Affiliations:** Department of Biochemistry, All India Institute of Medical Sciences, New Delhi, India; Department of Biostatics, All India Institute of Medical Sciences, New Delhi, India; Department of Emergency Medicine, JPNATC, All India Institute of Medical Sciences, New Delhi, India

## Abstract

**Introduction:**

The susceptibility to adverse outcome from critical injury (occurrence of sepsis, septic shock, organ dysfunction/failure, and mortality) varies dramatically due to different degrees of inflammatory response. We assessed the relationship of the genotype distribution of various cytokine gene polymorphisms (CGP) with regard to the development of sepsis, organ dysfunction or mortality in severely injured patients.

**Method:**

Observational, hospital-based cohort study of 114 severely injured North Indian patients from New Delhi admitted to the Emergency Department (ED) of Trauma Centre, AIIMS. Patients were monitored from day first to discharge or death, measuring SOFA score, sepsis and septic shock occurrences up to one month. We have analyzed 13 cytokine genes, including the SNPs of structural and regulatory regions at 22 positions.

**Results:**

Sequence-specific primer based PCR indicated that eight polymorphic loci IL-1α /-889, IL-1β/-511, IL-1R (pstI 1970), TGF-β/ code 10, TNF-α/-308, TNF-α/-238, IL-6/+565 and IL-10/-1082, out of 22 SNPs are significantly associated with sepsis morbidity and outcome. Theses SNPs might be used as risk determinants of the outcome. Patients with IL-10 (−1082A/A) genotypes were found significantly higher in post traumatic sepsis patients and had a significantly higher risk to developed sepsis complication (*p* < 0.05, OR = 0.86, C.I = 0.08-8.8).In case of TNF-α (−308) position, GA and GG genotype patients have a significantly lower risk of poor outcome (*p* < 0.05, OR = 0.25, C.I = 0.01-1.3) and (*p* < 0.05, OR = 0.22, C.I = 0.01-0.5) in comparison to AA genotype. In this study, two polymorphisms (IL-1β (−511) and IL-1R) were significantly associated with the development of MOF and mortality, where as IL-1α (−889) polymorphism associated with susceptibility for sepsis. The distribution of haplotypes of TGF-β and IL-6 were also associated with sepsis susceptibility and outcome.

**Conclusion:**

In conclusion, we have found that the alternations in the genotype and allele frequency of IL-1β (−511C/T), TNF-α (−308 G/A), TNF-α (−238 G/A) and IL-10 (−1082 G/A) genes are associated with an higher risk of sepsis development in trauma patients and outcomes.

**Electronic supplementary material:**

The online version of this article (doi:10.1186/s13049-015-0174-3) contains supplementary material, which is available to authorized users.

## Introduction

Despite tremendous advancements in pre-hospital system and treatment in hospitals, trauma emerged as the fourth leading cause of death among all diseases worldwide [1, 2]. Posttraumatic sepsis as a result of hysterical immune inflammatory responses is one of the leading sequential dysfunction of vital organs and most commonly seen problems associated with the ICU [3, 4]. Though there have been many advances in the development of antibiotics and caring care, sepsis remains a solemn and deadly problem with high mortality rates worldwide [5]. Therefore, prognostic markers to identify high-risk patients are immediately needed for early detection and preventive care of sepsis. Cytokines play vital roles in the regulation of host immune response, and distorted expression of cytokines are proven to be involved the development of sepsis [6]. Previous studies suggests that variations in the genes encoding cytokines are also involved in the inflammatory responses and are responsible for inter-individual differences in susceptibility to sepsis and its severity [7, 8]. Delineating the variations in cytokine genes and associated differences in response to sepsis might contribute to the development of new genetically modified diagnostic and therapeutic interventions that may improve outcome in post traumatic sepsis patients. According to the biphasic model of trauma etiology, derangement of both pro-inflammatory as well as anti-inflammatory immune responses eventually culminates into sepsis associated mortalities [9].

Reasons associated with the above mentioned conditions are under meticulous investigations, but numerous studies has advocated the significance of genetic variations (particularly single nucleotide polymorphisms) as key determinants of inter-individual variations in both inflammatory responses and clinical outcome in trauma patients [10, 11] . Single nucleotide polymorphism (SNPs) are the key factors that regulate the expressional variation of human genes and found to be associated with the susceptibility and progression [12]. Studies of TNF-α gene polymorphisms at position −308 and −238 have been reported within the promoter region, and showed susceptibility and resistance between population [7, 13]. Interestingly, IL-6 (−174 G/C) polymorphism influenced the immune pathogenesis of sepsis in European populations of trauma [14]. Ryan et al. (2013) studied the cytokine gene polymorphism and outcome after traumatic brain injury in population of the United Kingdom and observed polymorphism associated with resistance to sepsis and outcome [15]. Polymorphisms at IL-10 promoters:-1082 (G/A), −819 (C/T) and −592 (C/A) are associated with resistance to sepsis in Caucasians population [16, 17] . Interleukin (IL-1) gene complex polymorphism associated susceptibility for sepsis was studied in trauma patients of Chinese population [7, 8].

Contemplating it, we evaluated the polymorphisms in fallowing cytokine genes; interleukin IL-1-α (T/C-889), IL-1-β (−511 C/T, T/C + 3962), IL-1RA (T/C mspal1100), IL-4RA (G/A + 1902), IL-12 (C/A-1188), IFN-γ (+874 A/T), TGF-β1 (C/T codon10, G/C codon25), TNF-α (G/A −308, G/A −238), IL-2 (T/G −330 G/T +166), IL-4(T/G-1098, T/C-590, T/C-33), IL-6 (G/C −174, G/A nt 560), IL-10 (G/C −1082, C/T −819, C/A −592) in trauma hemorrhagic shock (THS) patients with respect to their secretary levels. The major goal of this study: (1) To find out cytokine gene polymorphism found in trauma patients who had developed sepsis with non-sepsis as well as survivors with non-survivors to unravel their role in the predisposition of disease progression, (2) to evaluate the SNP-SNP interaction of cytokine gene polymorphisms with respect to their expression in serum of trauma patients.

We observed significant association of TNF-α ^-238 (AA)^, IL-1α^-889 (CC)^ and IL-10-1082^(GG)^ genotype in sepsis and IL-1β ^-511 (CT)^, IL-1R^pstI1970 (TT)^ and TGFβ ^codon25 (GG)^ genotype in non-survivors. Interestingly, these genotypes are present in trauma patients, thus shedding some light on the predisposition to the progression of sepsis and also from non-sepsis to sepsis. However,TNF-α ^-238(AA)^, IL-1α^-889(CC)^ and IL-10-1082^(GG)^ genotypes appeared to be a higher producer by CBA assay, but IL-10 masked the expression of the high producing genotype of TNFα ^-238(AA)^ in sepsis patients. Expressions masking of TNF-α was found to be linked with the degree of IL-10 presence in sepsis patients.

## Materials and methods

### Patient evaluation and ethics

This study was approved by the institute ethics committee/ethics sub-committee of All India Institute of Medical Sciences, New Delhi, India *(Ref.No.IESC/T-358/30.09.2011)*. Patients of either sex (16–60 years old) with systolic blood pressure (SBP ≤90 mm Hg), olingourea or no urine output, presenting within the 8 h of trauma were recruited after taking the consent from the patient’s or their relatives. Those patients who had neurogenic shock, traumatic brain injury (TBI), septic shock and already resuscitated with anti-inflammatory drugs or corticosteroids before reporting to the Emergency Department (ED) were excluded from this study.

Patients in whom blood sampling was not initiated within the first 8 h after a trauma were also excluded from this analysis. Severity was assessed by Injury severity score (ISS) and Shock Index (S.I) in the ED and development of organ dysfunction was assessed by the Sequential Organ Failure Assessment score (SOFA; range, 0–24). Mortality was defined as death occurring till 30 days after the onset of hemorrhagic shock. Patients were classified as survivor if they were discharged alive from the ICU. Further, we have categorized the patients into two broad categories, those who developed sepsis complication and other those who did not during the 30 days of observational period.

All patients requiring surgical intervention received standard surgical care and postoperative intensive care unit treatment. Blood samples were drawn within 8 h after admission (designated day 0) and immediately used for DNA extraction for PCR-SSP assay.

### Sample collection

#### Collection of first blood sample

All patients were managed according to the protocol of the American College of Surgeons Advanced Trauma Life Support (ATLS). Patients were stabilized (SBP = 105 mmHg) with crystalloids or colloids before collecting the blood sample, which is collected on the spot in the Emergency Department (Day 0).

Blood samples were also collected from 140 healthy, age matched, uninjured individuals who formed the control group for DNA extraction and CGP analysis after taking the proper consent form from the patients.

### Definitions, scoring of severity and outcome

Abbreviated injury scale (AIS) score of traumatic injuries induced while living and diagnosed by autopsy were determined using the 1985 protocol of the American Association for Automotive Medicine [18]. AIS scores range from 1 to 6; total AIS scores, which represent the sum of scores for each body region (head and neck, face, chest, abdomen, extremities and external) and the ISS [18], which is defined as the sum of the square of the single highest AIS score in each of the three most severely injured body regions were determined for each case. Shock Index (S.I) is the ratio of Heart rate and Systolic blood pressure (SBP) were calculated for every patient in ED to understand the extent of hemorrhagic shock.

The SOFA score is used to track a patient’s status during the stay in the ICU. The SOFA score is a scoring system to determine the extent of a person’s organ function or rate of failure [3, 19]. The score is based on six different scores, one each for the respiratory, cardiovascular, hepatic, coagulation, renal and neurological systems. Emphasizing on the potential prognostic value of the serum cytokines, evaluated the correlation with the injury severity and SOFA score.

### Systemic Inflammatory Response Syndrome (SIRS)

The systemic inflammatory response to a wide variety of severe clinical insults, manifested by two or more of the following conditions: (1) Temperature > 38 °C or < 36 °C, (2) Heart rate > 90 beats/min, (3) Respiratory rate > 20 breaths/min or PaCO_2_ < 32 mmHg, (4) WBC count > 12,000/mm^3^, < 4000/mm^3^, or > 10 % immature (band) forms.

### Sepsis

The systemic inflammatory response to infection. In association with infection, manifestations of sepsis are the same as those previously defined for SIRS. It should be determined whether they are a direct systemic response to the presence of an infectious process and represent an acute alteration from baseline in the absence of other known causes of such abnormalities. The clinical manifestations would include two or more of the following conditions as a result of a documented infection.

### Multiple Organ Dysfunction Syndromes (MODS)

The presences of altered organ function in severe trauma patient such that homeostasis cannot be maintained without intervention.

### SNP selection

To investigate the genotype, we have selected IL-2, IL-12, IFN-γ, and TNF-α, IL-1α, IL-1β as pro-inflammatory cytokines and TGF-β, IL-4 and IL-10 as anti-inflammatory cytokine genes for studying genetic or SNP-SNP interaction. The receptor chain of IL-1R, IL-1Ra, IL-4R was also selected to find out the underlying functional discrepancy in the trauma patients. SNPs were selected according to the priority of minor allele frequency (5 %) publicity available database selected from the National Centre for Biotechnology Information (NCBI) Entrez SNP (build 36) and the International HapMap project: Han Chinese (Asian population). Few of the SNPs were included either on the basis of their functional role, as demonstrated or putative and well documented in the literature.

### Genotyping of SNPs for the cytokine genes

**Fig. 1:**
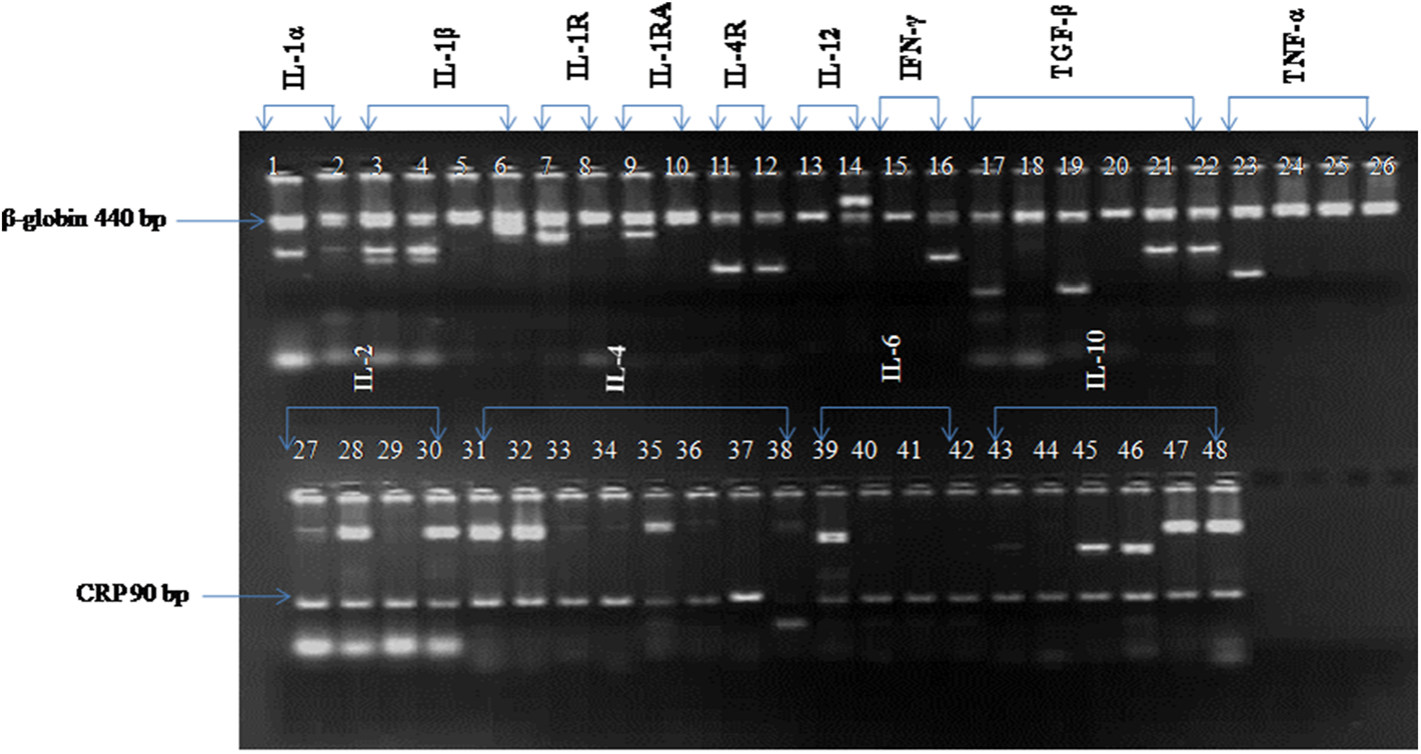
A representative 2 % agarose gel diagram of a battery of pro and anti-inflammatory cytokine gene variants. Each of the primer mixes contained a non-allelic internal control primer pair, which amplified either a part of the β-globin gene (440 bp amplicon) in upper row or a part of the C-reactive protein gene (CRP) at 89 bp in lower row

Total 10 mL venous blood from each trauma patients and healthy volunteers were collected. 5 mL of blood in EDTA vials was subjected to PCR-SSP assay, while another 5 ml collected with (no anticoagulant) was used for cytokine analysis by CBA. DNA was extracted by standard phenol-chloroform method, and stored at −80 °C, till further use. Samples showing 260/280 ratio as 1.82-1.86 were only used for the PCR-SSP analysis. Afterwards, above DNA was subjected to *in vitro* amplification using Cytokine CTS-PCR-SSP tray Kit (Heidelberg, Germany) according to manufacturer’s instructions. Each PCR tray of this kit is meant for two different samples. Each well contains two different primers: one for specific polymorphism and other for positive control. We run all the samples in duplicates. Amplified products were transferred onto 2 % agarose gel and electrophoresed at 100 V for 55 min to get the maximum resolution of polymorphic bands. As an internal positive control, a fragment of human globin gene (440 bp) for amplicons lanes containing IL-1α, IL-β, IL-1R, IL-4R, IL-12, IFN-γ, TGF-β and TNF-α (1–26 lanes of the gel) were co-amplified with a control of globin gene (440 bp) in the same well, while the lane containing amplicons of IL-2, IL-4 and IL-10 (27–48 lanes of the gel) were co-amplified with human C reactive protein gene (90 bp) as control. Representative gel picture for distribution of genotypes and alleles along with internal controls CRP gene (89 bp) and β-globulin gene (440 bp) are shown in (Fig 1).

### Statistical analysis

Statistical analyses were performed using STATA 11.2 (SPSS Inc., Chicago, IL, USA) and Graph Pad Prism 5.0 (Graph Pad Software, San Diego, CA, USA). Categorical data were compared using chi square (*χ*2) and continuous data were compared by the Mann–Whitney *U* test. Allele frequencies were obtained through direct counting. Odds ratio (OR) with 95 % confidence intervals (95 % CI) was also calculated using the same software. A *p*-value of <0.05 was considered statistically significant. Hardy-Weinberg equilibrium was used for each SNP employing Arle-quin software version 3.1. Univariate and Multivariable logistic regression was used to calculate the unadjusted and adjusted odds ratio for evaluating effects of genotype on mortality and sepsis.

## Results

### Outcome and clinical variables

Out of 130 patients enrolled in this study, 16 patients died within 24 h in the hospital were excluded from analysis. The age (mean ± SD) of 114 patients (96 male and 16 female) were 34.3 ± 14.5 years. Of the 114 patients, 77 (67.5 %) survived and 37 (32.5 %) were died after injury by one month (Table 1). The demographic and clinical data of 114 patients included in the study was shown in (Table 1) where as demographic and clinical data for the survivors and non-survivors were summarized in (Table 2). The age, gender and S.I were comparable between survivors and non-survivors. The SOFA and ISS score of non-survivors were significantly (*p* < 0.001) higher in comparison to survivors (Table 2). SOFA score of non-survivors were significantly (*p* < 0.001) higher with mean value 7.2 ± 2.3 and 10.3 ± 1.15 on day 7^th^ and 14^th^ respectively (Table 2). In this study, 25 patients (18 male & 7 female) developed sepsis within 30 days. The SOFA score, ISS score and gender were significantly differ between patients with sepsis and non-sepsis (Table 3).

**Table 1:** Clinical characteristics of trauma victim with hemorrhagic shock patients (*n* = 114)

Number	Percent	
Age (yrs), mean ± sd	34.3 ± 14.5	
	(16–60)	
Male/Female	98/16	
No of body region injured		
Two	52	45.6
Three	35	30.7
Four	27	23.6
ISS (Injury severity scale)	18.71 ± 8.48	
Shock Index (S.I)	1.17 ± 0.51	
Organ dysfunction		
One	35	30.7
Two	27	23.6
Three or above	12	10.5
Cause of trauma		
Traffic accident	82	71.9
Free fall from a height	17	14.9
Heavy weight blunts Injury	15	13.1
Survival	77	67.5
Death	37	32.5

**Table 2:** Clinical characteristics of survivors and non-survivors patients

	Survivors	Non-survivors	*P* value
Patients	*n* = 77	*n* = 37	
Sex (M/F)	65/12	33/4	0.35
Age (yrs)	33.7 ± 14.4	35.6 ± 14.8	0.26
ISS	15.2 ± 6.4	26.1 ± 7.3	0.02
S.I	1.14 ± 0.35	1.2 ± 0.74	0.43
SOFA-7	3.5 ± 1.57	7.2 ± 2.34	0.001
SOFA-14	5.2 ± 1.7	10.3 ± 1.15	0.001

M/F represents males and females. ISS and S.I represent Injury severity scale and Shock Index

SOFA-7 and SOFA-14 represents the sequential organ failure assessment score on day 7 and 14

**Table 3:** Clinical characteristics of patients with sepsis and non-sepsis

	Sepsis	Non-sepsis	*P* value
Patients	*n* = 25	*n* = 89	
Sex (M/F)	18/7	72/17	0.02
Age (yrs)	37.5 ± 16.1	33.4 ± 13.9	0.21
ISS	21.2 ± 6.8	18 ± 8.8	0.05
S.I	1.2 ± 0.44	1.16 ± 0.53	0.5
SOFA-7	5.28 ± 1.96	3.78 ± 2.34	0.006
SOFA-14	7.86 ± 2.18	4.77 ± 2.31	0.0001
Mortality			
n (%)	16(64)	21(29.5)	0.001

M/F represents males and females. ISS and S.I represent Injury severity scale and Shock Index

SOFA-7 and SOFA-14 represents the sequential organ failure assessment score on day 7 and 14

### Cytokine gene polymorphism in patients and controls

We have recruited the 114 homogeneous north Indian patients with hemorrhagic shock in this study, and measured the distribution of genotype and allele frequency and correlated with 140 north Indian healthy controls. There was no significant (p ˃ 0.05) changes in distribution of genotype and allele frequency between these groups Additional file 1: Table S1.

### Cytokine gene polymorphism in post traumatic sepsis vs. non-sepsis patients

In this study, 25 patients (18 male & 7 female) developed sepsis within 30 days. The distribution of genotype and allele frequency of cytokine genes that were not significant in two groups are shown in Additional file 2: Table S2 where as significantly observed distribution between these groups are shown in (Table 4). On univariate logistic regression analysis, the distribution of genotypes at positions IL-1α (−889 C/T), IL-4 (−1098 T/G), IL-10 (−1082 A/G), allele A of TNF-α (−238) and allele T of IL-10 (−889) were significantly (*p* < 0.05) predominant in sepsis (Table 4). The SOFA score, ISS score and gender were significant (*p* < 0.05) in these groups (Tables 2 and 3). Further, we calculated the adjusted odds ratio after adjusting for SOFA, ISS and gender using a multivariate logistic regression model in both groups (Table 5).

**Table 4:** Genotypic and allelic frequency distribution in patients with sepsis and non-sepsis

Cytokine gene polymorphism	Genotype	Non-sepsis	Sepsis	*p* value	OR (95 % CI)
		f (%)	f(%)		
		*n* = 89	*n* = 25		
IL-1α(−889)	CC	45(50.56)	21(84)		1
	TC	32(35.96)	3(12)	0.01	0.20(0.05–0.73)
	TT	12(13.48)	1(4)		0.17(0.02–1.4)
	C	122(68.5)	45(90)		1
	T	56(31.5)	5(10)	0.002	0.24(0.07-0.6)
TNF-α(−308)	AA	3(3.3)	2(8)		1
	GA	17(19.1)	1(4)	0.09	0.08(0.05–1.3)
	GG	69(77.6)	22(88)		0.47(0.07–3.0)
	A	23(13)	5(10)		1
	G	155(87)	45(90)	0.57	1.3(0.46–4.7)
TNF-α(−238)	GG	78(87.7)	19(76)		1
	GA	10(11.2)	4(16)	0.13	0.2 (0.13–2.8)
	AA	1(1.1)	2(8)		0.12(0.01–1.4)
	G	166(93.2)	42(84)	0.04	1
	A	12(6.8)	8(16)		2.6(0.87–7.5)
IL-4(−1098)	TT	66(74.1)	12(48)		1
	TG	21(23.6)	9(36)	0.006	2.3(0.87–6.3)
	GG	2(2.3)	4(16)		11(1.8–66.9)
	T	153(86)	33(82)	0.001	1
	G	25(14)	17(18)		3.1(1.4–6.8)
IL-10(−1082)	GG	5(5.7)	4(16)		1
	GA	33(37)	2(8)	0.01	0.07 (0.01–0.52)
	AA	51(57.3)	19(76)		0.46 (0.11–1.9)
	G	30(16.9)	10(20)	0.60	1
	A	148(83.1)	40(80)		0.8(0.34–2.0)
IL-10(−819)	CC	63(70.7)	16(64)		1
	CT	22(24.7)	6(24)	0.39	0.10 (0.03–3.0)
	TT	4(4.6)	3(12)		0.65 (0.14–2.9)
	C	148(83.1)	12(76)	0.001	1
	T	30(16.9)	31(24)		12.7(5.5–30.1)

**Table 5:** Distribution of genotypes in non-sepsis and sepsis patients using multivariate logistic regression analysis

Cytokine gene polymorphism	Genotype	Non-sepsis	Sepsis	Unadjusted odds ratio		Adjusted odds ratio	
		f (%)	f (%)	OR (95 % C.I)	*p* value	OR (95 % C.I)	*p* value
		*n* = 89	*n* = 25				
IL-α(−889)	CC	45(50.56)	21(84)	1		1	
	TC	32(35.96)	3(12)	0.20(0.05–0.73)	0.01	0.21(0.05–0.79)	0.02
	TT	12(13.48)	1(4)	0.17(0.02–1.4)	0.10	0.25(0.02–2.1)	0.20
TNF-α(−238)	GG	78(87.7)	19(76)	1		1	
	GA	10(11.2)	4(16)	0.2 (0.13–2.8)	0.2	0.19(0.01–3.1)	0.2
	AA	1(1.1)	2(8)	0.12(0.01–1.4)	0.09	0.09(0.007–1.3)	0.08
TNF-α(−308	AA	3(3.3)	2(8)	1		1	
	GA	17(19.1)	1(4)	0.08(0.05–1.3)	0.07	0.10(0.006–1.6)	0.10
	GG	69(77.6)	22(88)	0.47(0.07–3.0)	0.43	0.64(0.09–4.6)	0.66
IL-4(−1098)	TT	66(74.1)	12(48)	1		1	
	TG	21(23.6)	9(36)	2.3(0.87–6.3)	0.09	1.8(0.47–7.5)	0.36
	GG	2(2.3)	4(16)	11(1.8–66.9)	0.009	6.1(0.58–64)	0.12
IL-10(−1082)	GG	5(5.7)	4(16)	1		1	
	GA	33(37)	2(8)	0.07(0.01–0.52)	0.01	0.36(0.03–3.5)	0.38
	AA	51(57.3)	19(76)	0.65(0.11–1.9)	0.29	1.9(0.33–11.5)	0.45

n (%) = number of subjects (expressed as percentage),OR = odds ratio, CI = class intervals

Adjusted odds ratio for ISS, SOFA score and gender

The IL-1 gene polymorphism corresponding to IL-1α (−899), IL-β (−511) and IL-1β (+3962) were analyzed between patients with sepsis and non-sepsis. However, significant (*p* < 0.05) differences in distribution of genotype and allele frequency were observed for IL-1α (−899) position. At this position three genotypes CC, TC and TT were analyzed in both groups. The frequency of CC. TC and TT genotype in patients with sepsis and non-sepsis were 84 % vs. 50.5 %, 12 % vs. 36 % and 4 % vs. 13.4 % (*p* < 0.05) respectively (Table 4). Interestingly, we noticed patients with TC genotype had significantly lower risk of developing sepsis as compared to homozygous genotypes CC and TT (*p* = 0.01, OR (95 %) =0. 21, CI = 0. 05–0.79). The allele frequency of C among sepsis (90 %) was significantly higher as compared to non-sepsis (68.5 %, *p* = 0.002). However, the adjusted odd ratio TC genotype at this position were 0.21 times, lowered in comparison to CC genotype (*p* = 0.02, CI = 0.05-0.79) (Table 5).

The TNF-α gene has a biallelic polymorphism in the promoter region at position −308 and −238, representing G to A transitions were studied in both groups (Table 4). The genotype frequency at −238 position were 76 % vs. 87.7 % for AA, 16 % vs. 11.2 % for GA and 8 % vs. 1.1 % for GG in patients with sepsis and non-sepsis respectively (Table 4). On univariate logistic regression analysis, the allelic frequency of allele A were 2.6 times higher odd to develop sepsis (*p* = 0.04, OR (95 %) =2.6, CI = 0.87-7.5) compared to non-sepsis patients (Table 4). At position-308, the distribution of genotype frequency of GG, GA and AA were comparable between two groups (Table 5). The genotype frequency of GA in sepsis (4 %) was comparatively lower than non-sepsis (19.1 %) (*p* = 0.099, OR (95 %) =0.08 and CI = 0.05-1.3). At (−1098 T/G) position of the IL-4 gene, we observed that patients having GG genotypes had significantly (*p* = 0.009, OR (95 %) =11, CI = 1.8-66.9) higher risk to develop sepsis complications as compared to other two genotypes (TT and TG). The allelic frequency of allele G in sepsis (18 %) was found to be significantly (*p* = 0.001, OR (95 %) =3.1, CI = 1.4-6.8) higher as compared to non-sepsis (14 %) (Table 4).

Three well known SNPs were reported in IL-10 genes, two in promoter regions (−1082,-819) and another in coding region (+592). However, we observed skewed genotypes at −1082 positions only. The distribution of genotypes frequency of GG, GA and AA was found to be as 16 % vs. 5.7 %, 8 % vs. 37 % and 76 % vs. 57.3 % in patients with sepsis and non-sepsis respectively (Table 5). Patients with GA genotype had 0.07 times lower odd to developed sepsis [OR (95 % CI) :(0.01-0.52), *p* = 0.01]. Patients with allele T at IL-10 (−819) had 12.5 times more chances of developing sepsis as compared to non-sepsis (*p* = 0.001, OR (95 % CI) = 12.5, CI = 5.5-30.1).

The distribution of haplotype of the TGF-β, TNF-α, IL-2, IL-4, IL-6 and IL-10 genes were studied in patients with sepsis and non-sepsis (Table 6). However, the distribution of haplotypes in TNF-α and IL-10 genes were observed significant (*p* < 0.05) in both groups. The frequency of GA in patients with sepsis (8.0 %) was significantly higher as compared to non-sepsis (0 %) (*p* < 0.05). Other haplotypes GG and AG were comparable in both groups. Haplotype ATA of IL-10 gene in patients with sepsis (16 %) was significantly (*p* = 0.01) higher in comparison to non-sepsis (1.2 %). However, haplotype ACC was found to be predominant in both groups. Other haplotype ACC and GCC were observed similar in patients with sepsis and non-sepsis (Table 6).

**Table 6:** Distribution of haplotype in patient with sepsis and non-sepsis

Cytokine gene	Haplotypes	Distribution of haplotype in patients with sepsis and non-sepsis			
Polymorphism		Sepsis patients	Non-sepsis patients	*χ* ^2^	*p* value
		*n* (%)	*n* (%)		
TGF-β codon 10 C/T& codon25 G/C	CG	19(76)	72(80.9)		
	CC	2(8)	1(1.1)	3.6	0.18
	TG	4(16)	16(18.0)		
TNF-α-308 G/A &-238 G/A	GG	21(92)	86(96.7)		
	GA	2(8)	0(0)	8.0	0.05
	AG	0(0)	3(3.3)		
IL2 -330 T/G & + 160 G/T	GG	6(24)	18(20.2)		
	TG	19(76)	65(73)	1.8	0.76
	GT	0(0)	1(1.1)		
	TT	0(0)	5(5.7)		
IL4-1098 T/G, −590 C/T & -590 C/T	GCC	0(0)	2(2.2)		
	TCC	20(80)	70(78.6)	0.57	0.99
	TTT	5(20)	17(19.2)		
IL6-174 G/C & + 565 A/G	GG	24(96)	86(96.6)	0.50	0.63
	CG	0(0)	1(1.1)		
	GA	1(4)	2(2.3)		
IL10 -1082 G/A, −819C/T &-592 C/A	GCC	8(32)	30(33.7)		
	ACC	13(52)	58(65.1)	10.4	0.01
	ATA	4(16)	1(1.2)		

### Cytokine gene polymorphism in non-survivors and survivors patients

In this study, out of 114, 37(32.5 %) patients died within 30 days. On univariate logistic regression analysis, the genotypes IL-1R (pst I 1970 T/T), IL-1β (−511 C/T) and TGFβ (codon 25 G/G) and allele A of IL-12(−1188 C/A), allele G of IL-4R(+1902) and allele T of IL-1R were predominant in non-survivors (Table 7). The genotype, which was observed comparable in both groups of patients is shown in Additional file 3: Table S3. We found significantly higher SOFA and ISS score in non-survivors as compared to survivors (Tables 2 and 3). Further, we calculated the adjusted odds ratio after adjusting for SOFA and ISS score using a multivariate logistic regression model in both groups (Table 8). The distribution of CC, CT and TT genotype at IL-1β (−511) were observed significant (*p* = 0.03) in both groups (Table 7). The odds of finding of CT genotype was 2.7 times higher in non-survivors as compared to survivors (*p* = 0.03, OR (95 %) = 2.7 & CI = 1.0-7.1) (Table 7). The TT genotype of IL-1R (pst I1970) was significantly higher compared to non-survivors (*p* = 0.05, OR (95 %) =5.0, CI = 1.5-16.9) (Table 7). The allelic frequency of allele T in non-survivors (52.7 %) was significantly (*p* = 0.01, OR (95 %) =2.0 and CI = 0.62-2.5) higher as compared to patients with survivors (35.8 %) (Table 7).

**Table 7:** Comparison of genotypic and allelic frequency in non-survivors and survivors patients

Cytokine gene polymorphism	Genotype/Allele	Survivors	Non-survivors	*p* Value	OR (95 % CI)
		*n* (%)	*n* (%)		
		*n* = 77	*n* = 37		
IL-1β(−511)	CC	34(44.1)	9(24.4)		1
	CT	27(35)	20(54)	0.03	2.7(1.0–7.1)
	TT	16(20.9)	8(21.6)		1.8(0.61–5.8)
	C	95(83.3)	38(51.3)	0.13	1
	T	59(16.7)	36(49.7)		1.5(0.83–2.7)
IL-1R(pst I 1970)	CC	29(37.6)	9(24.3)		1
	CT	41(53.2)	17(45.9)	0.02	1.3(0.52–3.4)
	TT	7(9.0)	11(29.8)		5.0(1.5–16.9)
	C	99(64.2)	35(47.3)	0.01	1
	T	55(35.8)	39(52.7)		2.0(1.0–3.6)
IL-4R(+1902)	AA	51(66.2)	17(46)		1
	GA	22(28.6)	16(43)	0.08	2.1(0.93–5.0)
	GG	4(5.2)	4(11)		3(0.67–13.3)
	A	124(80.5)	15(20.2)	0.001	1
	G	30(19.5)	59(79.8)		16.2(7.7–34.7)
IL-12(−1188)	CC	10(12.9)	3(8.1)		1
	CA	25(32.4)	9(24.3)	0.49	1.2(0.26–5.3)
	AA	42(54.7)	25(67.6)		1.9(0.49–7.9)
	C	45(29.2)	47(20.2)	0.001	1
	A	109(70.8)	27(79.8)		0.23(0.12–0.44)
TGF-β(codon25)	CC	61(79.2)	26(70.27)		1
	CG	14(18.2)	8(21.62)	0.04	1.3(0.50–3.6)
	GG	2(2.6)	3(8.1)		3.5(0.55–22.3)
	C	136(88.3)	60(81.1)	0.14	1
	G	18(11.7)	14(18.9)		0.56(0.24–1.3)

**Table 8:** Distribution of genotypes in non-survivors and survivors patients using multivariate logistic regression analysis

Name of the cytokine gene with position	Genotype	Survivors	Non-survivors	Unadjusted odds ratio		Adjusted odds ratio	
		f (%)	f (%)	OR (95 % CI)	*p* value	OR (95 % CI)	*p* value
		*n* = 77	*n* = 37				
IL-1R(pst I 1970)	CC	29(37.6)	9(24.3)	1		1	
	CT	41(53.2)	17(45.9)	1.3(0.52–3.4)	0.54	0.9(0.06–14.8)	0.99
	TT	7(9.0)	11(29.8)	5.0(1.5–16.9)	0.08	19.9(0.6–577)	0.08
IL4R(+1902)	AA	51(66.2)	17(46)	1		1	
	GA	22(28.6)	16(43)	2.1(0.93–5.0)	0.07	3.6(1.0–12.4)	0.48
	GG	4(5.2)	4(11)	3(0.67–13.3)	0.14	21.8(3.2–148)	0.61
IL-1β(−511)	CC	34(44.1)	9(24.4)	1		1	
	CT	27(35)	20(54)	2.7(1.0–7.1)	0.03	9.8(0.75–128.2)	0.08
	TT	16(20.9)	8(21.6)	1.8(0.61–5.8)	0.26	3.2(0.09–114	0.51
TGF-β(codon25)	CC	61(79.2)	26(70.27)	1		1	
	CG	14(18.2)	8(21.62)	1.3(0.50–3.6)	0.55	1.7(0.50–6.4)	0.37
	GG	2(2.6)	3(8.1)	3.5(0.55–22.3)	0.18	15(1.5–141)	0.01

Adjusted odds ratio at 95 % CI of genotypes with ISS and SOFA score

At IL-4R position, AA genotype in survivors (66.2 %) was1.5 times higher compared to non-survivors (46 %) (*p* > 0.05). The allele G was (*p* = 0.001, OR (95 %) =16.2 and CI = 7.7-34.7) four folds (79.8 %), higher in non-survivors in comparison to survivors (19.5 %) (Table 7). At position −1188, the distribution of 12.9 % vs. 8.1 % for CC, 32.4 % vs. 24.3 % for CA and 54.7 % vs. 67.6 % for AA (*p* = 0.49) was observed in survivors and non-survivors patients (Table 7). The frequency of allele A in non-survivors (79.8 %) was significantly (*p* = 0.01, OR (95 %) =0.23 and CI = 0.12-0.44) higher as compared to survivors (70.8 %) (Table 7).

In case of TGF-β, two SNPs, one at codo10 and another at codon 25 were analysed in survivors and non-survivors patients. However, the distribution of genotype at codon 25 position was significant (*p* < 0.05) in both groups. At this position, genotype observed were 79.2 % vs. 70.2 % for CC, 18.2 % vs. 21.6 % for CG and 2.6 % vs. 8.1 % for GG in survivors and non-survivors respectively (Table 7). However, after adjusting odd ratio for SOFA and ISS score at this position, using multivariate logistic regression analysis, the frequency of GG genotype was significantly (*p* = 0.01, OR(95 %) = 15 and CI = 1.5-141) higher in non-survivors as compared with survivors (Table 8).

Moreover, the distribution of haplotype of the TGF-β, TNF-α, IL-2, IL-4, IL-6 and IL-10 genes were studied in survivors and non-survivors (Table 9). A significant, distribution of haplotypes in TGF-β gene was observed significant in both groups. The haplotype frequency of CT in non-survivors (8.1 %) was significantly (*p* = 0.02) higher in comparison to survivors (0 %) patients.

**Table 9:** Distribution of Haplotype frequency in non-survivors and survivors patients

Cytokine gene	Haplotypes	Distribution of haplotype in survivors and non-survivors patients			
Polymorphism		Survivors	Non-survivors	*χ* ^2^	*p* value
		*n* (%)	*n* (%)		
TGF-β codon 10 C/T& codon25 G/C	CG	65(84.4)	26(70.2)		
	CC	0(0)	3(8.1)	7.3	0.02
	TG	12(15.6)	8(21.7)		
TNF-α-308 G/A &-238 G/A	GG	75(97.4)	34(91.9)		
	GA	1(1.3)	1(2.7)	1.9	0.22
	AG	1(1.3)	2(5.4)		
IL2 -330 T/G & + 160 G/T	GG	18(23.3)	6(16.2)		
	TG	55(71.4)	19(78.4)	3.2	0.37
	GT	0(0)	1(2.7)		
	TT	4(5.2)	1(2.7)		
IL4-1098 T/G, −590 C/T & -590 C/T	GCC	1(1.3)	1(2.7)		
	TCC	64(83.1)	26(70.3)	2.5	0.25
	TTT	12(15.6)	10(27)		
IL6-174 G/C & + 565 A/G	GG	74(96.1)	36(97.3)		
	CG	1(1.3)	0(0)	0.48	0.99
	GA	2(2.6)	1(2.7)		
IL10 -1082 G/A, −819C/T &-592 C/A	GCC	26(33.7)	12(32.4)		
	ACC	47(61.1)	24(64.8)	0.42	0.99
	ATA	4(5.2)	1(2.8)		

### Estimation of serum cytokine levels in sepsis, non-sepsis patients and healthy controls

Elevated levels of IL-1α, TNF-α and IL-10 were observed in patients with sepsis compared to healthy controls and non-sepsis patients (Table 10). CC genotype was the higher producer of IL-1α compared to other genotypes (Table 10). IL-1α was higher in the serum of sepsis (5.4 ± 2.8 vs. 18.6 ± 7.8 pg/ml, HC vs. sepsis, *p* < 0.05) than non-sepsis (5.4 ± 2.8 vs. 7.2 ± 2.6 pg/ml, HC vs. non-sepsis). Notably, a significantly higher level of TNF-α was found in sepsis patients (25.7 ± 18.6 vs. 57.6 ± 18.7 pg/ml, HC vs. sepsis, *p* < 0.001) than non-sepsis (25.7 ± 18.6 vs. 37.5 ± 16.8 pg/ml, HC vs. non-sepsis, *p* < 0.0001). The AA genotype of TNF-α (−308) and GA genotype of TNF-α (−238) were found as the higher producer of cytokines. Interestingly, GG genotype of IL-10 (−1082) was predominant in sepsis patients and higher producer (11.6 ± 4.7 vs. 84.5 ± 37.5 pg/ml, HC vs. sepsis, *p* < 0.001).

**Table 10:** Showing the serum levels of IL-1α, TNF-α and IL-10 in healthy controls and patients

Genotype		Healthy controls		Non-sepsis		Sepsis		*p* Value
		Percent	pg/ml	Percent	pg/ml	Percent	pg/ml	
IL-1α(−889)	CC	48.5	5.4 ± 2.8	50.5	7.2 ± 2.6	84	18.6 ± 7.8	NS, p^b<0.05^, p^c<0.05^
	CT	38.6	5.7 ± 1.9	35.9	8.3 ± 3.1	12	21.5 ± 8.7	NS, p^b<0.05^, p^c<0.05^
	TT	12.9	3.7 ± 2.7	13.6	6.7 ± 2.7	4	16.2 ± 9.2	p^a<0.05^, p^b<0.05^, p^c<0.05^
TNF-α(−308)	AA	2.8	25.7 ± 18.6	3.3	37.5 ± 16.8	8	57.6 ± 18.7	p^a<0.05^, p^b<0.001^, p^c<0.001^
	GA	17.2	15.4 ± 9.7	19.1	34.6 ± 13.5	4	54.7 ± 19.6	p^a<0.001^, p^b<0.001^, p^c<0.001^
	GG	80	11.2 ± 7.2	77.6	32.5 ± 12.7	88	51.2 ± 16.3	p^a<0.05^, p^b<0.001^, p^c<0.0001^
TNF-α(−238)	GG	90	27.6 ± 16.7	87.7	35.2 ± 14.6	76	40.6 ± 23.5	p^a<0.05^, p^b<0.001^, p^c<0.0001^
	GA	8.5	18.5 ± 12.3	11.2	30.5 ± 13.4	16	62.8 ± 18.6	p^a<0.05^, p^b<0.001^, p^c<0.0001^
	AA	1.5	14.8 ± 7.8	1.1	32.4 ± 12.7	8	38.8 ± 14.8	p^a<0.05^, p^b<0.001^, p^c<0.0001^
IL-10(−1082)	GG	7.1	11.6 ± 4.7	5.7	21.6 ± 13.5	16	84.5 ± 37.5	p^a<0.01^, p^b<0.0001^, p^c<0.0001^
	GA	18.6	14.7 ± 5.2	37	22.5 ± 15.6	8	74.6 ± 26.3	p^a<0.01^, p^b<0.001^, p^c<0.0001^
	AA	74.3	9.2 ± 4.5	57.3	16.7 ± 11.2	76	67.8 ± 21.4	p^a<0.01^, p^b<0.001^, p^c<0.0001^

*p* value (*p* < 0.05) considered as significant. It is calculated by two tailed Mann–Whitney, p^a^(healthy controls vs. non-sepsis),p^b^(healthy controls vs sepsis) and p^c^(sepsis vs. non-sepsis)

## Discussion

Genetic susceptibility of sepsis and the patients at high risk of disease progression are still a matter of debate. Deciphering genes involved in the host susceptibility or resistance towards sepsis in trauma patients will allow researchers to redefine the principles of pathogenesis of sepsis and outcomes. Trauma patients present a state where genetic variations in cytokine production dictate various immune reactions and as a result, patients correspond with different clinical outcomes. The present study was conducted to explore the variation in cytokine signature and susceptibility of post-trauma sepsis and outcome.

The complexity of pathogenesis and the highly polymorphic nature of the inflammatory response as well as genome make it extremely unlikely that an arbitrarily selected candidate SNPs will prove to functionally significant. To this end, we hypothesized that groups of some SNPs in cytokine genes may work together to influence the clinical outcome of major trauma patients by regulating the cytokines levels. Under purview of this hypothesis, we selected the 13 cytokines gene and 22 SNPs which are not only common polymorphism in North Indian population, but also functional variations expected to affect the outcome [20].

The main finding of this study is that SNPs of key cytokines showed susceptibility to sepsis in trauma patients, clinical outcomes and mortality that can be used as markers of predisposition to sepsis. As for clinical relevance, eight polymorphic loci IL-1α/-889, IL-1β/-511, IL-1R (pstI 1970), TGF-β/ codon 25, TNF-α/-308, TNF-α/-238, IL-6/+565, and IL-10/-1082 have been shown to be significantly associated with sepsis morbidity and outcome that may be used as a determinant of risk factor for the outcome of trauma patients. Thus, trauma patients were stratified into different groups based on carriage of 22 SNPs and the above 8 risk SNPs, respectively. There existed significant interaction among SNPs in relation to the development of sepsis and organ dysfunction in trauma patients in two cases of analysis. The present study shows statistically significant association between the genotype of TNF-α and the development of sepsis and outcome. Subjects homozygous for the allele A of TNF (−238) showed significantly higher risk for the development of sepsis and have more chance of death in the ICU. Compared to other studies, our findings lead to similar results concerning the frequency distribution of genotype and allele at TNF-α (−238) SNPs [15, 21, 22]. Patients having heterozygous for the allele TNF-308 G/A had a lower risk of developing sepsis. In confirmation of this finding, we also observed the distribution of haplotype at this position that are produced due to combinations of genotypes-308 G/A and −238 G/A. We observed those patients who had G to A transition (G to A) had significantly higher risk of developing sepsis and also had elevated serum levels of TNF-α [23].

Interleukin-1 (IL-1) family is a critical mediator of immune response to sepsis with two agonists (IL-1α and IL-1β) and one antagonist (IL-1 receptor antagonist; IL-1RA) [20, 24]. In this study, two polymorphisms (IL-1β (−511) and IL-1R) were significantly associated with the development of MOF and mortality where as IL-1α (−889) polymorphism was associated with susceptibility for sepsis. The results of our study revealed that individuals with CC genotype at IL-1β (−511) had more chances of developing MOF and also showed higher mortality as compared to individuals of TT and CT. In contrast, no association was observed for sepsis at this position. This work is also supported by various studies in mouse model of trauma, Caucasian and Chinese population that indicated that IL-1β polymorphism are associated with many diseases including inflammatory bowel disease, rheumatoid arthritis and gastric cancer [7, 24, 25]. To avoid inference from possible gender and severity dependent differences in outcome following trauma, adjusted odds ratio was calculated after adjustment of gender, ISS and SOFA in sepsis and non-sepsis patients. The heterozygous (TC) genotypes of IL-1 (−889) showed independent risk factor for the susceptibility of sepsis in trauma patients.

We have found an association between the IL-4 (−1098 T/G) polymorphism and susceptibility for sepsis and outcomes. Similarly, IL-4R (+1902 A/G) polymorphism and mortality was observed, but it failed to correlate towards susceptibility for sepsis. The IL-4 gene polymorphism and susceptibility of various diseases like asthma and malaria were studied in Caucasian population [26–28], but susceptibility of sepsis in trauma patients was showed by limited number of studies [27]. Moreover, we can’t conclude that the association of IL-4 and IL-4R polymorphism was linked with an unfavorable prognosis of the syndrome because the functional relevance of such variants on cytokine production is still unknown.

High level of IL-10 secretion may be associated with poor outcome after trauma, and identification of those patients likely shows sensitivity to infection, may enable novel targeted therapy in near future. Interestingly, we noticed trauma patients who had (G to A) substitution at IL-10(−1082) positions were protective for the sepsis and produced significantly lower level of IL-10 in comparison to GG genotype patients. Long et al. [9] demonstrated that the −1082 A allele was significantly associated with sepsis development after major trauma. The study by Long et al. and other studies has clearly demonstrated that the −1082 A/G polymorphism in the IL-10 gene promoter has an important impact on susceptibility to sepsis and outcome. In contrast, we did not observe significant difference for association between the −819 and −592 polymorphism of IL-10 gene. The present study also observed that the ATA haplotype was significantly lower in sepsis patients in comparison to non-sepsis. The gene encoding IL-10 cytokine involved in the modulation of inflammatory responses as a main anti-inflammatory agent is therefore a candidate gene for determination of the human genetic background, which is responsible for inter individual differences in susceptibility to sepsis development.

The predominance of high producer genotype of TNF-α with sepsis patients are not clear in this study. Interestingly, the varied amount of TNF-α in all three groups: healthy controls, non-sepsis and sepsis patients showed an example of expression masking of the genotype with a high producer genotype of IL-10 in these patients. In other words degree of TNF-α in sepsis patients is associated with the production of IL-10. To avoid inference from possible gender and severity dependent differences in outcome following trauma, adjusted odds ratio was calculated after adjustment of ISS and SOFA in survivors and non-survivors patients. The GG genotype of TGF-β (codon 25) was observed significantly three folds higher in non-survivors (8.1 %) in comparison to survivors (2.6 %) after adjusted with ISS and SOFA score. The distribution of genotypes at codon 25 and functional relevance was not observed in this study. However, the functional relevance of TGF-β gene polymorphism and their relevance in the suseptability to Helicobacter pylori related disease in known [29].

This study demonstrates to acknowledge for the first time from India, that the genotype and alleles of the cytokine genes is associated with susceptibility and outcome. The IL-1β, TNF-α and IL-10 polymorphism frequency was significantly higher in the post traumatic septic group than in the non-sepsis and appeared to be an independent risk factor for death due to septic shock. We are aware that relatively small sample size limits our study; a type II error may have occurred during analysis. Moreover, selection bias cannot be excluded; even though no differences of age, sex and ISS between groups were observed, such selection bias may have contributed to differences in cytokine levels at baseline and have affected association results. Thus, it is not surprising that the functional significance of some SNPs was not observed. Nonetheless, the most significant and intriguing finding of our study still remains the significant combined effect of cytokine gene SNPs on clinical outcome of major trauma patients.

## Conclusion

In conclusion, our study demonstrates that the alternations in the genotype and allele frequency of IL-1β (−511C/T), TNF-α (−308G/A), TNF-α (−238G/A) and IL-10(−1082G/A) genes are associated with an increased risk of sepsis development in major trauma patients and outcomes. These associations support the hypothesis that an increase in levels of IL-β, TNF-α and IL-10 secretion due to genetic variation in their genes may contribute to the development of sepsis after severe trauma by affecting the induced immune response. The results of this study need to be confirmed in a large-scale prospective study from some other ethnic populations, with encouraging further to develop novel, perhaps individually-tailored, anti-inflammatory therapies for the prevention of sepsis development after major trauma.

### Key message

1. This study demonstrates to acknowledge for the first time from India, that the genotype and alleles of the cytokine genes are associated with susceptibility for sepsis and outcome.
2. As for clinical relevance, eight polymorphic loci IL-1α /-889, IL-1β/-511, IL-1R (pstI 1970), TGF-β/ codon 10, TNF-α/-308, TNF-α/-238, IL-6/+565, and IL-10/-1082 have been shown significantly associated with sepsis morbidity and outcome and might be used as risk determinant’s for the outcome of trauma patients.
3. Our study showed an increase in levels of IL-β, TNF-α and IL-10 secretion due to genetic variation in their genes may contribute to the development of sepsis after severe trauma by affecting the induced immune response.
4. The present study also observed that the ATA haplotype of the IL-10 gene and CC haplotype of TGF-β gene was significantly associated with sepsis and outcome of trauma patients.

## Additional files

Additional file 1: Table S1. Cytokine gene polymorphism in THS patients and controls. (DOCX 20 kb) Additional file 2: Table S2. Genotypic and allelic frequency distribution in patients with sepsis and non-sepsis. (DOCX 19 kb) Additional file 3: Table S3. Comparison of genotypic and allelic frequency in non-survivors and survivors patients. (DOCX 20 kb)

## Abbreviations

ISS: Injury severity score
MOF: Multiple organ failure
S.I: Shock Index
SIRS: Systemic Inflammatory response syndrome
SOFA: Sequential Organ Failure Assessment score
THS: Trauma hemorrhagic shock
SASS: Sepsis and septic shock
