# Supplementary material for: Clinical relevance of single nucleotide polymorphisms within the 13 cytokine genes in North Indian trauma hemorrhagic shock patients
Source: Scand J Trauma Resusc Emerg Med. 2015 Nov 11;23:96. doi: 10.1186/s13049-015-0174-3 (PMC4642631; doi:10.1186/s13049-015-0174-3)
Supplement: Additional file 1: Table S1. — Cytokine gene polymorphism in THS patients and controls. (DOCX 20 kb) [file 13049_2015_174_MOESM1_ESM.docx]

**Table S1: Cytokine gene polymorphism in THS patients and controls**

| Cytokine gene polymorphism | Genotype  /Allele | Hemorrhagic shock patient, n(%) | Healthy controls n(% | χ^2^ | p value |
| --- | --- | --- | --- | --- | --- |
| IL-α(-889) | CC  TC  TT  C  T | 66(57.9)  35(30.7)  13(11.4)  167(73.2)  61(26.8) | 68(45.3)  54(38.6)  18(12.9)  190(67.8)  90(32.2) | 2.2  1.7 | 0.34  0.18 |
| IL-1β(-511) | CC  CT  TT  C  T | 43(37.7)  47(41.2)  24(21.1)  133(58.3)  95(41.7) | 30(21.4)  82(58.5)  28(20.1)  142(50.7)  138(49.3) | 3.4  2.9 | 0.09  0.08 |
| IL-1β(+3962) | CC  TC  TT  C  T | 80(70.2)  16(14.0)  18(15.8)  176(78)  50(22) | 74(52.9)  6(4.2)  60(42.9)  154(55)  126(45) | 4.6  0.19 | 0.19  0.66 |
| IL-1R(pst I 1970) | CC  CT  TT  C  T | 38(33.4)  58(50.8)  18(15.8)  134(58.8)  94(41.2) | 46(32.9)  82(58.6)  12(8.5)  174(62.1)  106(37.9) | 3.4  0.60 | 0.17  0.43 |
| IL-1RA(MSPAL11100) | CC  TC  TT  C  T | 3(2.6)  31(27.2)  80(70.2)  37(16.2)  191(83.8) | 2(1.4)  50(35.7)  88(62.9)  52(18.5)  228(81.5) | 2.4  0.48 | 0.28  0.48 |
| IL-4R(+1902) | AA  GA  GG  A  G | 68(59.7)  38(33.3)  8(7.0)  174(76.3)  54(23.7) | 70(50)  52(37.1)  18(12.9)  192(68.5)  88(31.5) | 3.4  3.7 | 0.17  0.06 |
| IL-12(-1188) | CC  CA  AA  C  A | 13(11.4)  34(29.8)  67(58.8)  60(26.3)  168(73.7) | 24(17.2)  44(31.4)  72(51.4)  92(32.8)  188(67.2) | 2.0  2.5 | 0.34  0.60 |
| INF-γ(874) | AA  AT  TT  A  T | 57(50)  39(34.2)  18(15.8)  153(67.1)  75(32.9) | 60(42.9)  44(31.4)  36(25.7)  164(58.5)  116(41.5) | 3.7  3.9 | 0.15  0.06 |
| TGF-β(codon10) | CC  CT  TT  C  T | 37(32.4)  58(50.9)  19(16.7)  132(57.8)  96(42.2) | 28(21.4)  76(54.3)  36(24.3)  136(48.5)  144(51.5) | 3.3  3.8 | 0.08  0.06 |
| TGF-β(codon25) | CC  CG  GG  C  G | 89(78.0)  22(19.4)  3(2.6)  200(87.7)  28(12.3) | 118(84.3)  18(12.8)  4(2.9)  254(90.7)  26(9.3) | 2.5  0.24 | 0.26  0.09 |
| TNF-α(-308) | AA  GA  GG  A  G | 3(2.6)  18(15.7)  93(81.7)  24(10.5)  204(89.5) | 4(2.8)  24(17.2)  112(80)  32(11.4)  248(88.6) | 0.48  0.51 | 0.80  0.47 |
| TNF-α(-238) | AA  GA  GG  A  G | 2(1.7)  14(12.2)  98(86.1)  18(7.8)  210(92.2) | 29(1.4)  12(8.5)  126(90)  70(25)  210(75) | 1.4  1.7 | 0.47  0.08 |
| IL-2(-330) | TT  TG  GG  T  G | 12(10.5)  79(69.3)  23(20.2)  103(45.1)  125(54.9) | 30(21.5)  98(70)  12(8.5)  162(57.8)  118(42.2) | 4.7  5.2 | 0.17  0.12 |
| IL-2(+160) | GG  GT  TT  G  T | 83(72.8)  26(22.8)  5(4.4)  192(84.2)  36(15.8) | 88(62.8)  40(28.6)  12(8.6)  216(77)  64(23) | 2.5  0.02 | 0.27  0.88 |
| IL-4(-1098) | TT  TG  GG  T  G | 82(71.9)  30(26.3)  2(1.8)  194(85)  34(15) | 110(78.6)  28(20)  2(1.4)  248(88.5)  32(11.5) | 4.9  4.9 | 0.08  0.06 |
| IL-4(-590) | CC  TC  TT  C  T | 87(76.3)  24(21.0)  3(2.7)  198(86.8)  30(13.2) | 106(75.7)  32(22.8)  2(1.5)  244(87.1)  36(12.9) | 0.55  0.01 | 0.77  0.92 |
| IL-4(-33) | CC  TC  TT  C  T | 93(81.6)  19(16.7)  2(1.7)  205(89.9)  23(10.1) | 106(75.7)  20(14.3)  14(10)  232(82.8)  48(17.2) | 7.2  5.2 | 0.06  0.08 |
| IL-6(-174) | GG  GC  CC  G  C | 80(70.2)  28(24.6)  6(5.3)  178(81.6)  40(18.4) | 112(80)  28(20)  0(0)  252(90)  28(10) | 8.7  7.2 | 0.13  0.07 |
| IL-6(+565) | GG  GA  AA  A  G | 93(81.6)  18(15.8)  3(2.6)  204(90.6)  24(9.4) | 110(78.6)  26(18.6)  4(2.8)  246(87.8)  34(12.2) | 0.36  0.32 | 0.88  0.56 |
| IL-10(-1082) | GG  GA  AA  G  A | 8(7.0)  35(30.7)  71(62.3)  51(22.3)  177(77.7) | 10(7.1)  26(18.6)  104(74.3)  46(16.4)  234(83.6) | 5.1  2.8 | 0.07  0.09 |
| IL-10(-819) | CC  CT  TT  C  T | 79(69.3)  28(24.6)  7(6.1)  186(81.5)  42(18.5) | 90(64.3)  30(21.4)  20(14.3)  210(75)  70(25) | 2.8  3.6 | 0.11  0.07 |
| IL-10(592) | CC  CA  AA  C  A | 45(39.5)  53(46.5)  16(14.0)  143(62.7)  85(37.3) | 66(47.1)  54(38.6)  20(14.3)  186(66.4)  94(33.6) | 1.7  0.76 | 0.40  0.38 |
