# Supplementary material for: Clinical relevance of single nucleotide polymorphisms within the 13 cytokine genes in North Indian trauma hemorrhagic shock patients
Source: Scand J Trauma Resusc Emerg Med. 2015 Nov 11;23:96. doi: 10.1186/s13049-015-0174-3 (PMC4642631; doi:10.1186/s13049-015-0174-3)
Supplement: Additional file 2: Table S2. — Genotypic and allelic frequency distribution in patients with sepsis and non-sepsis. (DOCX 19 kb) [file 13049_2015_174_MOESM2_ESM.docx]

**Table S2. Genotypic and allelic frequency distribution in patients with sepsis and non-sepsis.**

| Cytokine gene polymorphism | Genotype | Non-sepsis  f (%)  n=89 | Sepsis  f(%)  n=25 | p value | OR  (95% CI) |
| --- | --- | --- | --- | --- | --- |
| IL-1β(-511) | CC  CT  TT  C  T | 30(33.7)  39(43.8)  20(22.4)  99(55.6)  79(44.4) | 13(52)  8(32)  4(16)  34(68)  16(32) | 0.27  0.11 | 1  0.47(0.17-1.2)  0.46(0.13-1.6)  1  0.58(0.28-1.1) |
| IL-1β(+3962) | CC  TC  TT  C  T | 64(71.91)  11(12.36)  14(15.73)  139(78)  39(22) | 16(64)  5(20)  4(16)  37(74)  13(13) | 0.56  0.54 | 1  1.8(0.55-5.9)  1.1(0.33-3.9)  1  1.2(0.55-2.7) |
| IL-1R(pst I 1970) | CC  CT  TT  C  T | 31(31.83)  45(50.56)  13(14.61)  107(60)  71(40) | 7(28)  13(52)  5(20)  27(54)  23(46) | 0.72  0.43 | 1  1.2(0.45-3.5)  1.7(0.45-6.3)  1  1.2(0.64-2.5) |
| IL-1RA(MSPAL11100) | CC  TC  TT  C  T | 2(2.25)  25(28.09)  62(69.66)  29(16.2)  149(83.8) | 1(4)  6(24)  18(72)  8(16)  42(84) | 0.83  0.96 | 1  0.47(0.03-6.2)  0.58(0.04-6.7)  1  1.0(0.41-2.7) |
| IL-4R(+1902) | AA  GA  GG  A  G | 52(58.43)  31(34.83)  6(6.74)  135(75.8)  43(24.2) | 16(64)  7(28)  2(8)  37(74)  13(26) | 0.76  0.78 | 1  0.73 (0.27-1.9)  1.0(0.19-5.8)  1  1.1(0.49-2.3) |
| IL-12(-1188) | CC  CA  AA  C  A | 10(11.24)  23(25.84)  56(62.92)  43(24.1)  135(75.9) | 3(12)  11(44)  11(44)  17(34)  33(66) | 0.18  0.16 | 1  1.5(0.36-6.9)  0.65(0.15-2.7)  1  0.6(0.30-1.3) |
| INF-γ(874) | AA  AT  TT  A  T | 46(51.69)  28(31.46)  15(16.85)  120(67.4)  58(32.6) | 11(44)  11(44)  3(12)  33(66)  17(34) | 0.58  0.85 | 1  1.6(0.62-4.2)  0.83(0.20-3.4)  1  1.0(0.51-2.1) |
| TGF-β(codon10) | CC  CT  TT  C  T | 31(34.83)  43(48.31)  15(16.85)  105(58.9)  73(41.1) | 6(24)  15(60)  4(16)  27(54)  23(46) | 0.56  0.52 | 1  1.8(0.62-5.1)  1.3(0.33-6.2)  1  1.2(0.61-2.4) |
| TGF-β(codon25) | GG  CG  CC  G  C | 70(78.6)  16(17.9)  3(3.5)  156(87.6)  22(12.4) | 17(68)  6(24)  2(8  40(80)  10(20) | 0.37    0.16 | 1  1.5(0.54-4.6)  8.4(0.78-9.9)  1  1.7(0.68-4.2) |
| IL-2(-330) | GG  TG  TT  G  T | 17(19.1)  64(71.9)  8(8.99)  98(55)  80(45) | 6(24)  15(60)  4(16)  27(54)  23(46) | 0.37  0.9 | 1  0.66 (0.22-1.9)  1.4 (0.31-6.4)  1  1.0(0.52-2.0) |
| IL-4(-590) | CC  TC  TT  C  T | 68(76.4)  19(21.3)  2(2.3)  155(87)  23(23) | 19(76)  5(20)  1(4)  43(86)  7(14) |  | 1  0.94 (0.31-2.8)  1.7 (0.15-20.8)  1  1.0(0.37-2.8) |
|  |  |  |  | 0.80  0.84 |  |
| IL-4(-33) | CC  TC  TT  C  T | 72(80.9)  16(17.9)  1(1.2)  160(89.8)  18(10.2) | 21(84)  3(12)  1(4)  45(90)  5(10) | 0.45  0.98 | 1  0.64 (0.17-2.4)  3.4 (0.20-57.1)  1  0.98(0.27-2.9) |
| IL-6(-174) | GG  GC  CC  G  C | 63(70.9)  22(24.7)  4(4.4)  148(85.4)  30(14.6) | 17(68)  6(24)  2(8)  40(88)  10(12) | 0.77  0.60 | 1  1.1 (0.3-2.6)  1.8(0.31-10.9)  1  1.2(0.49-2.8) |
| IL-6(+565) | GG  GA  AA  G  A | 71(79.7)  16(17.9)  2(2.6)  158(88.7)  20(11.3) | 22(88)  2(8)  1(4)  46(92)  4(8) | 0.37  0.51 | 1  0.40 (0.08-1.8)  1.6 (0.13-18.6)  1  0.68(0.16-2.1) |
| IL-10(592) | CC  CA  AA  C  A | 37(41.5)  38(42.7)  14(15.8)  112(62.9)  66(37.1) | 8(32)  15(60)  2(8)  31(62)  19(38) | 0.33  0.90 | 1  1.8 (0.69-4.8)  0.66 (0.12-3.4)  1  1.0(0.51-2.0) |
