# Supplementary material for: Clinical relevance of single nucleotide polymorphisms within the 13 cytokine genes in North Indian trauma hemorrhagic shock patients
Source: Scand J Trauma Resusc Emerg Med. 2015 Nov 11;23:96. doi: 10.1186/s13049-015-0174-3 (PMC4642631; doi:10.1186/s13049-015-0174-3)
Supplement: Additional file 3: Table S3. — Comparison of genotypic and allelic frequency in non-survivors and survivors patients. (DOCX 20 kb) [file 13049_2015_174_MOESM3_ESM.docx]

**Table S3. Comparison of genotypic and allelic frequency in non-survivors and survivors patients.**

| Cytokine gene polymorphism | Genotype  /Allele | Survival  n (%)  n=77 | Death  n (%)  n=37 | p Value | OR (95% CI) |
| --- | --- | --- | --- | --- | --- |
| IL-α(-889) | CC  TC  TT  C  T | 43(55.9)  24(31.1)  10(13)  110(71.5)  44(28.5) | 23(62.2)  11(29.7)  3(8.1)  57(77.1)  17(22.9) | 0.70  0.37 | 1  0.85(0.35-2.0)  0.56(0.14-2.2)  1  0.74(0.36-1.4) |
| IL-1β(+3962) | CC  TC  TT  C  T | 55(71.4)  11(14.2)  11(14.4)  121(78.5)  33(21.5) | 25(67.5)  5(13.5)  7(18)  55(74.3)  19(25.7) | 0.78  0.47 | 1  1(0.31-3.1)  1.4(0.48-4.0)  1  1.2(0.62-2.5) |
| IL-1RA(MSPAL11100) | CC  TC  TT  C  T | 2(2.5)  24(31.2)  51(62.3)  28(18.1)  126(81.9) | 1(2.7)  7(18.9)  29(78.4)  9(12.1)  65(87.9) | 0.33  0.24 | 1  0.58(0.04-7.4)  1.1(0.09-13.08)  1  1.6(0.68-4.0) |
| INF-γ(874) | AA  AT  TT  A  T | 40(51.9)  26(33.7)  11(14.4)  106(68.8)  48(31.2) | 17(45.9)  13(35.13)  7(18.91)  47(63.5)  27(36.5) | 0.73  0.42 | 1  1.1(0.49-2.8)  1.4(0.49-4.5)  1  1.2(0.67-2.3) |
| TGF-β(codon10) | CC  CT  TT  C  T | 24(31.2)  41(53.3)  12(15.5)  89(57.8)  65(42.2) | 13(35.2)  17(45.9)  7(18.9)  43(58.1)  31(41.9) | 0.76  0.96 | 1  0.76(0.31-1.8)  1.0(0.34-3.4)  1  0.98(0.53-1.7) |
| TNF-α(-308) | AA  GA  GG  A  G | 2(2.6)  11(14.2)  64(83.2)  15(9.7)  139(90.3) | 3(8.1)  7(18.9)  27(73.0)  13(13.5)  61(86.5) | 0.29  0.09 | 1  0.42(0.05-3.2)  0.28(0.04-1.7)  1  0.50(0.21-1.2) |
| TNF-α(-238) | AA  GA  GG  A  G | 2(2.6)  12(15.6)  63(81.8)  14(9.1)  138(90.9) | 1(2.7)  2(5.4)  34(91.9)  4(5.4)  70(94.6) | 0.24  0.32 | 1  0.33(0.01-5.6)  1.0(0.09-12.3)  1  1.77(0.53-7.6) |
| IL-2(-330) | GG  TG  TT  G  T | 17(22)  53(69)  7(9.0)  87(56.5)  67(43.5) | 6(16.2)  26(70.3)  5(13.5)  38(51.4)  36(48.6) | 0.59  0.46 | 1  1.3(0.49-3.9)  2.0(0.46-8.8)  1  1.2(0.67-2.2) |
| IL-2(+160) | GG  GT  TT  G  T | 57(74)  16(20.9)  4(5.1)  130(84.4)  24(15.4) | 26(70.3)  10(27)  1(2.7)  62(83.7)  12(16.3) | 0.67  0.90 | 1  1.3(0.54-3.4)  0.43(0.04-3.8)  1  1.0(0.44-2.3) |
| IL-4(-1098) | TT  TG  GG  T  G | 56(72.7)  17(22.0)  4(5.3)  129(83.7)  25(16.3) | 22(59.4)  13(35.1)  2(5.5)  57(77)  17(23) | 0.33  0.21 | 1  1.9(0.81-4.6)  1.2(0.21-7.4)  1  1.5(0.71-3.2) |
| IL-4(-590) | CC  TC  TT  C  T | 60(77.9)  14(18.18)  3(3.90)  134(91.5)  20(8.5) | 27(72.97)  10(27.03)  0(0)  64(86.4)  10(13.6) | 0.33  0.91 | 1  1.5(0.6-4.0)  -  1  1.0(0.41-2.5) |
| IL-4(-33) | CC  TC  TT  C  T | 66(85.71)  9(11.69)  2(2.6)  141(91.5)  13(8.5) | 27(72.97)  10(27.03)  0(0)  64(86.4)  10(13.6) | 0.09  0.23 | 1  2.7(0.9-7.4)  -  1  1.6(0.62-4.4) |
| IL-6(-174) | GG  GC  CC  G  C | 55(71.4)  19(24.6)  3(3.9)  129(83.7)  25(16.3) | 25(67.5)  9(24.3)  3(8.2)  59(79.7)  15(20.3) | 0.60  0.45 | 1  1.04(0.41-2.6)  2.2(0.41-11.6)  1  1.38(0.59-2.8) |
| IL-6(+565) | GG  GA  AA  G  A | 64(83.2)  11(14.2)  2(2.6)  139(90.3)  15(9.7) | 29(78.4)  7(18.9)  1(2.7)  65(87.8)  9(12.1) | 0.81  0.57 | 1  1.4(0.49-3.9)  1.1(0.09-12.6)  1  1.2(0.46-3.3) |
| IL-10(-1082) | GG  GA  AA  G  A | 8(10.3)  22(28.5)  47(61.2)  38(20.1)  116(79.9) | 1(2.7)  13(35.1.7)  23(62.2.3)  15(14.8)  59(85.2) | 0.38  0.46 | 1  4.7(0.52-42.1)  3.9(0.46-33.2)  1  1.2(0.63-2.7) |
| IL-10(-819) | CC  CT  TT  C  T | 51(66.2)  20(26)  6(7.8)  122(79.2)  32(20.8) | 28(75.67)  8(21.63)  1(2.7)  64(86.4)  10(13.6) | 0.51  0.18 | 1  0.72(0.28-1.8)  0.30(0.03-2.6)  1  0.6(0.24-1.3) |
| IL-10(592) | CC  CA  AA  C  A | 29(37.6)  37(48)  11(14.4)  95(61.6)  59(38.4) | 16(43.3)  16(43.2)  5(13.5)  48(64.8)  26(35.2) | 0.89  0.64 | 1  0.78(0.33-1.8)  0.82(0.24-2.7)  1  0.87(0.46-1.6) |
